# Functional impacts of polyaniline in composite matrix of photocatalysts: an instrumental overview

**DOI:** 10.1039/d3ra01243c

**Published:** 2023-05-22

**Authors:** Joshua Akinropo Oyetade, Revocatus Lazaro Machunda, Askwar Hilonga

**Affiliations:** a School of Materials, Energy, Water and Environmental Science, Nelson Mandela African of Institution of Sciences and Technology PO Box 447 Arusha Tanzania joshuaoyetade@gmail.com oyetadej@nm-aist.ac.tz

## Abstract

The challenges associated with photocatalysts including their agglomeration, electron–hole recombination and limited optoelectronic reactivity to visible light during the photocatalysis of dye-laden effluent make it necessary to fabricate versatile polymeric composite photocatalysts, and in this case the incredibly reactive conducting polyaniline can be employed. The selection of polyaniline among the conducting polymers is based on its proficient functional impacts in composite blends and proficient synergism with other nanomaterials, especially semiconductor catalysts, resulting in a high photocatalytic performance for the degradation of dyes. However, the impacts of PANI in the composite matrix, which result in the desired photocatalytic activities, can only be assessed using multiple characterization techniques, involving both microscopic and spectroscopic assessment. The characterization results play a significant role in the detection of possible points of agglomeration, surface tunability and improved reactivity during the fabrication of composites, which are necessary to improve their performance in the photocatalysis of dyes. Accordingly, studies revealed the functional impacts of polyaniline in composites including morphological transformation, improved surface functionality, reduction in agglomeration and lowered bandgap potential employing different characterization techniques. In this review, we present the most proficient fabrication techniques based on the *in situ* approach to achieve improved functional and reactive features and efficiencies of 93, 95, 96, 98.6 and 99% for composites in dye photocatalysis.

## Abstract 2

The functional impacts of polyaniline in the fabricated photocatalyst nanocomposites used in dye photocatalysis were critically investigated *via* spectroscopic and microscopic instrumental overview.

## Graphical abstract

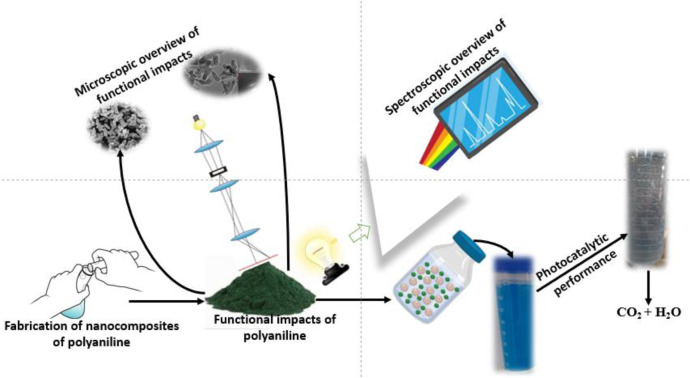

## 1 Introduction

The fabrication of nanocomposite photocatalysts for the photocatalysis of dyes in industrial effluents involves the active blending of two or more nanomaterials to form composites with multiple properties that are required for efficient photon capture and degradation of dyes in effluents associated with significant environmental toxicity.^1–3^ In this case, materials such as metals (Fe, Ag, Au, Sn, Wn, Ni, Pb, Co, *etc.*), metal oxide semiconductors (TiO_2_, ZnO, Cu_2_O, SiO_2_ Nb_2_O_5_, Fe_2_O_3_, FeO, WnO_3_, *etc.*), polymeric materials and other semiconductors such as graphene oxide, reduced graphene oxide, 2D-hexagonal boron nitride and carbon-based nanomaterials have been used independently and blended as nanocomposite photocatalysts.^3–7^ However, these materials exhibit major drawbacks of agglomeration, sensitivity to visible light and frequent electron–hole recombination in single and composite photocatalysts. Thus, to address these issues, the conducting polymer polyaniline (PANI) has been incorporated in the blend.^8,9^ Some other examples of conjugated conducting polymers are polyacetylene, polypyrrole, poly(thiophene), poly(*para*-phenylene vinylene) and poly(carbazole).^10–12^ Among them, the recent emphasis on PANI is based on its incredible morphological, reactive and functional impacts upon its incorporation in the fabrication of photocatalyst composites, enhancing their spontaneous degradative activities in the photocatalysis of recalcitrant dyes, which are present in large quantities in effluents.^13^ The impacts of PANI in the blend have the potential of tackling challenges of agglomeration and electron–hole recombination, which are major setbacks in photocatalysis.^9,12^ Furthermore, the PANI functionally is superior to other conducting polymers due to its unique charge transport dynamics, which accounts for its high photon-sensitizing impacts, while equally enhancing the sorption activity on fabricated composites.^14,15^ However, accessing the functional impacts of PANI during the fabrication of composites requires intensive microscopic and spectroscopic elucidation.^16,17^ A critical investigation employing microscopic and spectroscopic techniques gives vital information on the structural, functional, elemental and reactive effects as a result of the incorporation of PANI in the blend. The obtained information indicates the morphological modification and other alterations that influence the electron–hole recombination and agglomeration during photocatalysis.^18,19^ Also, the use of multiple microscopic and spectroscopic techniques can enable the fabrication of well-engineered photocatalyst composites with improved and ideal performances in the dye photocatalytic process. Thus, the informative readouts from these instruments suggest proficient pathways for the fabrication of improved photocatalyst composites.^16,17^ Fundamentally, the spectroscopic characterization of fabricated composites is related to the energy difference between the molecular energy levels of the composite catalysts under electromagnetic radiation.^20^ Alternatively, microscopic characterization with emphasis on electron microscopy (EM) provides vital information on the morphological impact of PANI in composite catalysts.^16,21^ These instruments enable the investigation of the point of possible set-backs during the fabrication of composites and the impacts of PANI regarding surface adjustment, bandgap lowering, surface functionalities and particle size together with the proficiency of the fabrication technique, which are vital features of ideal photocatalyst composites.^22–24^ Therefore, in this review, we critically investigate the functional impacts of polyaniline in the fabrication of nanocomposite photocatalyst, their properties and performance in dye photocatalysis *via* instrumental overview.

## 2 Synthesis and mechanistic action of PANI composites in dye photocatalysis

### 2.1 Synthesis mechanism of PANI

**Fig. 1:**
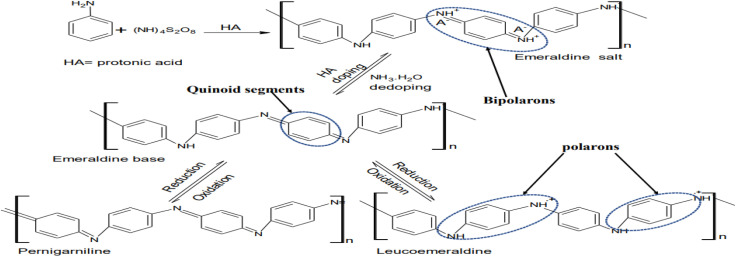
Mechanism for the synthesis of PANI.

**Fig. 2:**
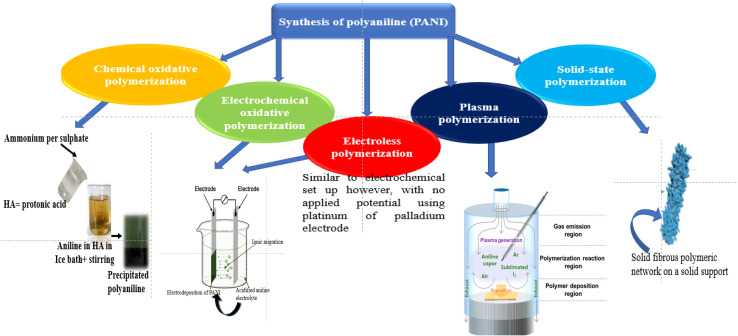
Various synthetic routes for polyaniline.

**Fig. 3:**
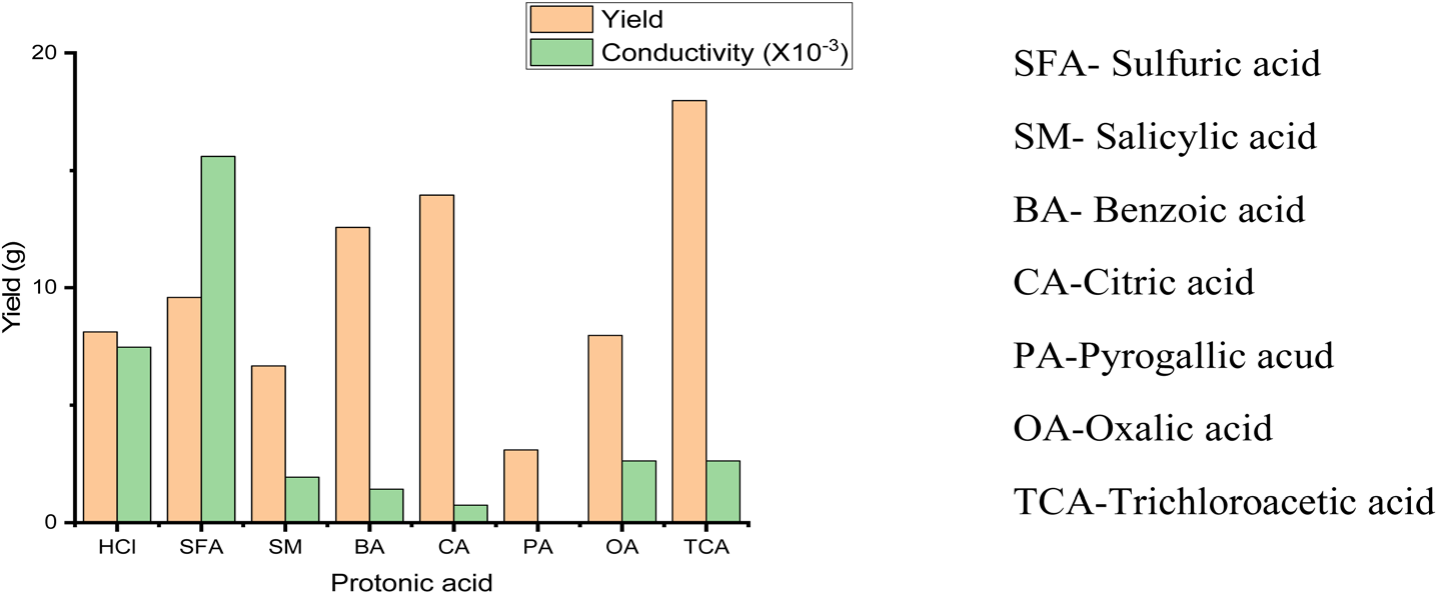
Yield and conductivity of synthesized polyaniline with respect to protonic acid by Motlatle *et al.*^155^.

Structurally, polyaniline (PANI) consists of a well-ordered structure of benzoid and quinoid functional groups, which is commonly synthesized *via* the oxidative polymerization of aniline in acid with ammonium persulphate (APS) to form leucoemeraldine, emeraldine or pernigraniline (Fig. 1).^4,25^ However, among its forms, the emeraldine homopolymer has the highest electron mobility, exceptional charge transport and lower band gap during photon irradiation.^5^ The other synthetic routes for this conducting macromolecule are described in Fig. 2. Besides oxidative polymerization, Fig. 2 indicates the use of an electrochemical oxidative route involving the application of an electrical current on the electrodes in the electrolyte (aniline in an acidic medium) of the electrothermal set-up.^26^ The applied current results in the electrochemical deposition of monomers on the oxidized positively charged electrode, leading to the deposition of the polymeric film.^27,28^ The advantage of this synthetic technique is its ability to control the desired parameters such as time, working temperature and solvent (acid dopant), which influence the morphology of the synthesized polymer.^29^ It should be noted that the choice of dopants used for the synthesis of this polymer results in a variation in its yield and electron transport dynamics, and consequently its conductivity (Fig. 3). In contrast to the above-mentioned route, the plasma polymerization route is initiated *via* ionization/excitement of the monomeric precursor, leading to the effective collision of monomeric molecules with a plasma-generated electron from the glow discharge of RF.^29^ Furthermore, electroless polymerization is similar to the electrochemical process but its novelty is the use of an electrochemical set-up without the application of an external potential for the deposition of PANI using specified inert electrodes such as platinum or palladium.^30^

Generally, based on instrumental characterization, the various synthetic routes of PANI result in distinct mechanical properties, which suggests its application as a network or polymeric support for other materials especially during the fabrication of composite photocatalysts with desired porosity.^26^ Also, the optoelectronic properties of the synthesized PANI are reflected in its incredible charge transport dynamics as a result of the polarons and bipolarons in its polymeric backbone together with the nitrogen of the protonated imine group.^31^ For instance, the emeraldine salt exhibits three distinct bands, *i.e.*, a band at 330 nm (π–π* transitions) and two other bands in the visible region at 430 nm (π → polaron band) and 800 nm (polaron → π*), having photon capture potential in the photocatalysis of dyes upon irradiation in the UV or visible spectrum.^26^

### 2.2 Photocatalytic mechanism

**Fig. 4:**
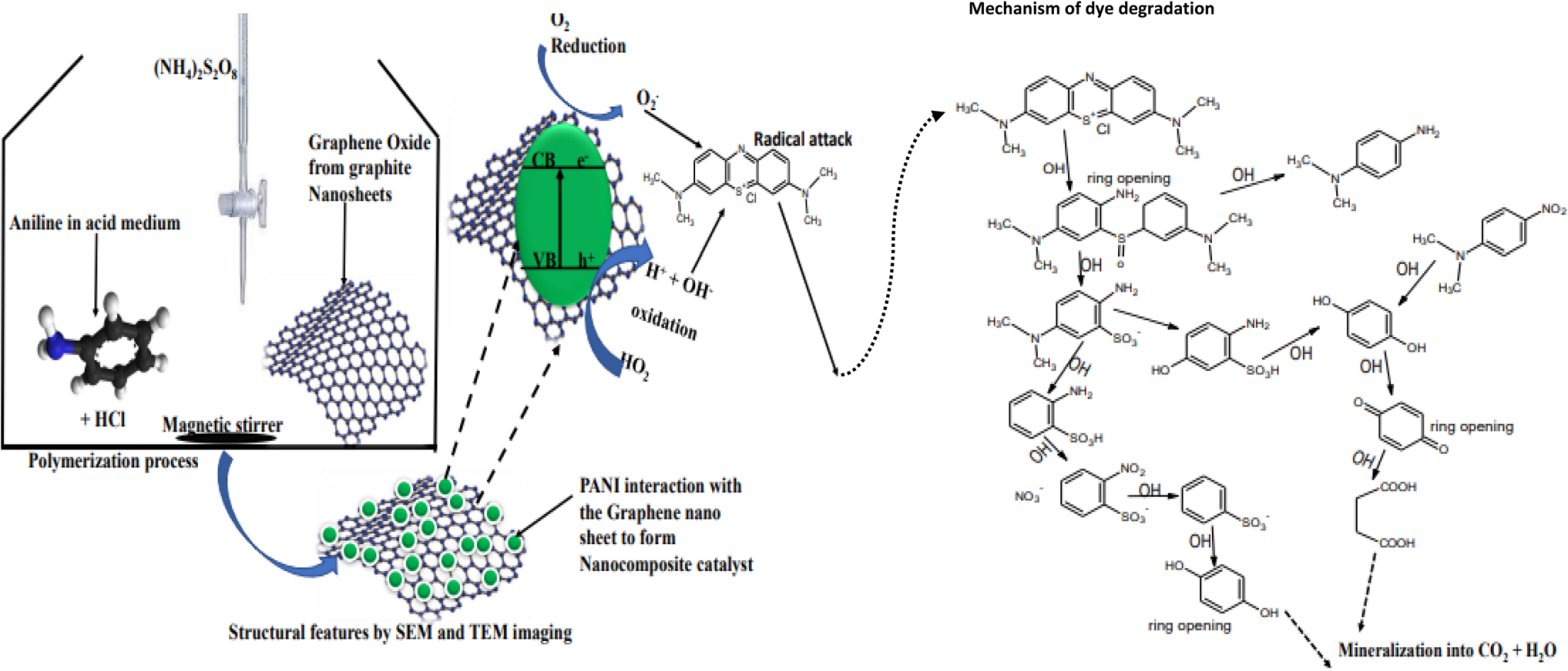
Synthesis of PANI composite and mechanism for the degradation of methylene blue dye.

Generally, photocatalysis is a photon-induced molecular transformation process that occurs at the surface of an excited photoactive nanomaterial (photocatalyst) that has adsorbed organic pollutants (*e.g.*, dye molecules) from the wastewater.^32^ This process entails a five-stage mechanism of photon capture, excitation of electrons from the valence band to the conduction band, generation of radicals such as hydroxyl radicals (˙OH), superoxide radical anions (˙O^2−^), and hydroperoxyl radicals (˙OOH) and radical attack, leading to the degradation of dye molecules (Fig. 4).^4,13,33^ Polyaniline and other materials in their pure or composite form follow this trend of generating radicals upon irradiation to mineralize adsorbed dye molecules into CO_2_ and H_2_O.^34^

### 2.3 Fabrication of PANI composite catalysts and mechanism in the process of dye photocatalysis

The fabrication of various composites of PANI for use in the photocatalytic degradation of dyes in effluent together with their merits and limitations is described in Table 1. Studies have classified nanomaterials that can be combined with PANI for the fabrication of composite photocatalysts as polymers, carbon-based materials, metals or metal oxide semiconductors.^4,35–38^ Among them, the emphasis on the use of semiconductors is based on their adsorption capacity, ability to undergo redox reactions during photoexcitation and morphological support.^33^ The capture of photons by these photocatalyst semiconductors leads to the generation of electron–hole pairs. However, they exhibit the limitations of electron–hole recombination, agglomeration and large band gap, influencing their photon capturing ability in the visible region. This has led to engineered fabrication with PANI to form composites.^39,40^

**Table 1:** Techniques for the fabrication of polyaniline composite catalysts

Fabrication technique	Description	Advantages	Limitations	Reference
Sonochemical	Polymerization of monomer with other materials, forming a composite under the impact of acoustic cavitation from ultrasonic irradiation	Effective mass transport	Requires sophisticated reactors	65
		Hight dispersion of particle-forming composites	Inefficient for large-scale production	
		Improved homogeneity of the mix	Low yield	
		Environmentally friendly	The problem of energy efficiency	
		Requires no additives		
		Shorter duration		
		High energy and pressure within a short time		
*In situ*	Polymerization of monomer blended in a solution with another nanomaterial simultaneously	Production of composites with higher interfacial strengths	Complicated steps and techniques	66
		Occurrence of simultaneous polymerization and composite fabrication	Difficulty in knowing the yield of PANI after polymerization	
Sol–gel	It is a two-stage sequential process involving the formation of the colloidal solution by hydrolysis (sol) before gelation (gel)	Simple	May require a toxic organic solvent	67
		Cost-effective	Long duration	
		Production of high-purity grade material	Possibility of contraction of material during processing	
			Residual hydroxyl of carbon group	
Emulsion and inverse emulsion	Incorporation of surfactant to form micelles on the surface of the nanomaterial before polymerization with the monomer, while inverse emulsion involves emulsification of monomer using a nonpolar organic solvent	Ability to control size and structure by different surfactants	Requires a large amount of surfactants	68
		Synthesis of stabilized composites	Not suitable for materials with a high melting point	
		Formation of low viscous materials		
		Simple		
		Low energy cost		
Mixing	This is an *ex situ* polymerization approach involving the physical blending of materials *via* solvent with polymer solution under mechanical agitation	Simple and direct	Requires solvent	69
		Cost-effective	Difficult in solvent removal	
		Ability to control the process	Possibility of solvent contamination	
			Weak phase interaction	
Hydrothermal	Synthesis of polymeric composites through hydrolysis reactions at high temperatures of the various compounds directly in an autoclave	Versatile in the synthesis of unstable materials	High energy consumption	66
		Synthesis of high composite material	High equipment cost	
Electrospinning	It is electrostatic spinning involving the extrusion of microfibre composites through a micro-syringe pump or spinneret in the presence of an applied voltage	Very efficient for nanofibre	Requires high voltage	70
		Simple		
		Cost-effective		
		Adjustable fiber diameter		
Chemical vapour deposition	It involves the collaborative formation of composites by combining the organic synthesis of polymers in the liquid phase with the formation of a coating by other materials forming composites in the vapour phase	Very effective in the formation of thin films	Long duration	71
		Polymeric composites formed are free coatings	Energy-intensive	
		High deposition rate	Difficult in handling	
		Forms composites with appreciable mechanical strength		

The properties of high charge transport dynamics and electron delocalization of the polymer lower the bandgap, while serving as a macrostructural support for semiconductor materials.^41,42^ For instance, as shown in Fig. 4, the fabrication of composites consisting of PANI and reduced graphene oxide *via in situ* polymerization results in unique structural features with higher adsorption capacity and lower bandgap, hindering electron–hole recombination. During irradiation, the excited electron jumps form HOMO to LUMO through π–π* in the polymer, forming positively charged holes.^4,43^ However, due to the synergistic interaction of the composite constituents, as the electron returns to the HOMO for recombination, it jumps into the empty conduction band of the semiconductor, creating efficient charge separation and impeding electron–hole recombination.^4^

### 2.4 Functional impacts of PANI in the fabricated composite catalysts

**Fig. 5:**
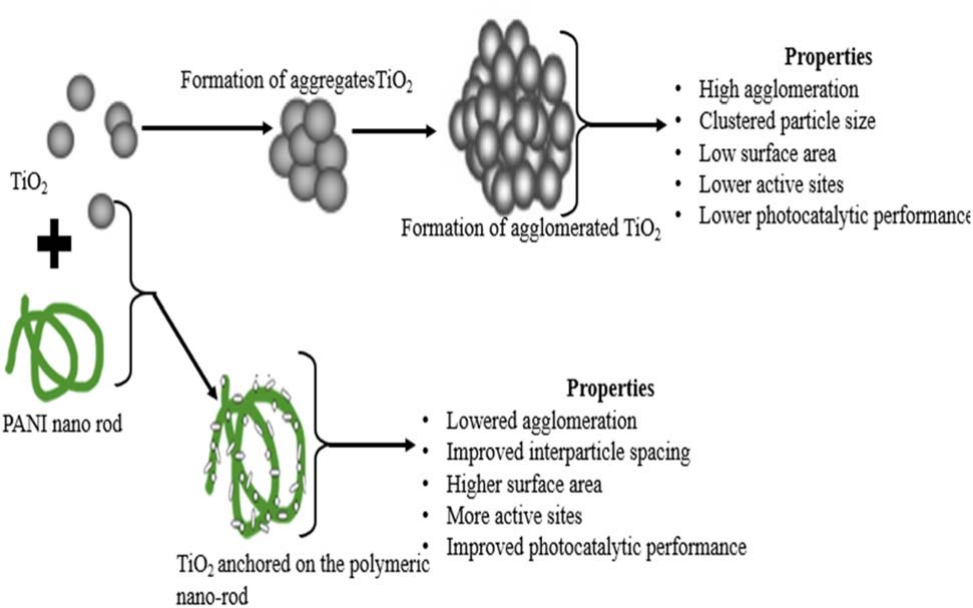
Mechanism of aggregation of TiO_2_ and its interaction with PANI.

Various pathways have been adopted for the fabrication of PANI-based composites (Table 1). Consequently, studies revealed that notable functional impacts arise from the incorporation of PANI in the composite blend, which gives the fabricated composite photocatalyst ideal features. One important feature is the decreased agglomeration of the formed nanoparticles, which is one of the main challenges associated with the application of photocatalysts in dye photocatalysis.^13^ The process of agglomeration of photocatalysts in nano form (10^−9^) involves the aggregation of the particles up to the point of adhesion to each other, forming a higher degree of agglomerates as a result of their higher surface energy (Fig. 5).^44,45^ This action limits the penetration of light, which is necessary for excitation in photocatalysis, and also results in the loss of surface area. A high surface area is desirable for the contact of the active site of the catalyst with the adsorbed dye molecules in the effluent.^46^ Alternatively, the introduction of PANI in the catalyst (TiO_2_, ZnO, Fe_2_O_3_, FeO, Ag_2_O, *etc.*) is based on the premise of anchoring the metal oxide nanoparticles on the macromolecular network of PANI, consequently reducing their agglomeration and appreciable interfacial distance (Fig. 5).^47,48^ For instance, a study of commercial TiO_2_ nanoparticles as photocatalysts revealed the formation of clustered particles with a low surface area (agglomerate). However, the introduction of PANI nanorods to form composites led to the encapsulation and uniform dispersion of the TiO_2_ nanoparticles on the surface of the conducting macromolecule (PANI).^49^ Furthermore, the spherical shape of TiO_2_, as reported by Zarrin and Heshmatpour,^8^ revealed the agglomeration of nanoparticles to form larger particles. However, the introduction of PANI with Nb_2_O_5_ resulted in the improved and uniform distribution of TiO_2_ on the polymeric network, thereby enhancing the reduction in particle aggregation.

Additionally, Yuan^50^ revealed that the large surface area of nanoparticles results in their aggregation, and consequently agglomeration. For instance, one of the commonly used semiconductors (graphene) exhibits a large surface area, resulting in the bundling of graphene sheets based on van der Waals forces.^51^ However, during the fabrication process, the incorporation of a polymer using procedures such as ultrasonication and surfactant and chemical modification improve the synergistic homogeneity of the constituent composite, thereby wrapping the semiconductors used around the polymer chain, leading to the formation of nanocomposite photocatalysts with less possibility of agglomeration.^50,52^ The impact of reduced agglomeration by the polymer equally improves the overall conductivity and imparts appreciative mechanical features in the composite.^52,53^

Additionally, it should be noted that another cause of agglomeration of catalysts in photocatalysis can arise from the use of excess catalyst during in the photocatalytic process of dye-laden effluent.^36,54^ Furthermore, the other impacts of PANI in the composite blend include improved optoelectronic features of the photocatalyst blend. This feature is vital and suggests the kinetics and responsive rate of the fabricated photocatalyst for use under irradiation with different photon sources (UV or visible) and intensities.^5,13^ The features define the combination of optical and electronic properties of the photocatalyst, including the bandgap value, excitation rate, and electron–hole recombination rate during photocatalysis.^55,56^ Most semiconductors used are affected by their high bandgap, affecting their sensitivity to photons from visible irradiation, which are required for higher performance in dye mineralization.^57,58^ Interestingly, the incorporation of PANI to form a nanocomposite photocatalyst significantly lowers both the bandgap and rate of electron–hole recombination.^4,59^ This impact is based on the unique π-conjugated electron systems of the conducting polymer, leading to proficient electron mobility.^5^ In addition, the attributes originating from the protonated nitrogen in the imine group and the well-ordered polymer chain with high conjugation produce unique electron mobility *via* an incredible hopping mechanism.^60^ However, it is worth noting that the various fabrication techniques highlighted in Table 1 play a significant role in features such as internal stresses and overall mechanical features of the materials in the composite system, which indicates their respective photocatalytic performance and recovery for reuse.^61,62^ Also, conditions such as the categories of nanofillers/semiconductors used, dispersion conditions, stirring rates and mixing ratio equally influence the thermal, mechanical and optoelectronic contribution of polyaniline in the blend.^13,61^ These functional impacts of the polymer in the fabrication process account for its extensive applications beyond photocatalysts to use in sensor fabrication based on its sensitivity to pH, while having appreciable thermal stability. Also, the resilience impact of PANI in the fabrication of films holds great future prospects^63,64^

**Fig. 6:**
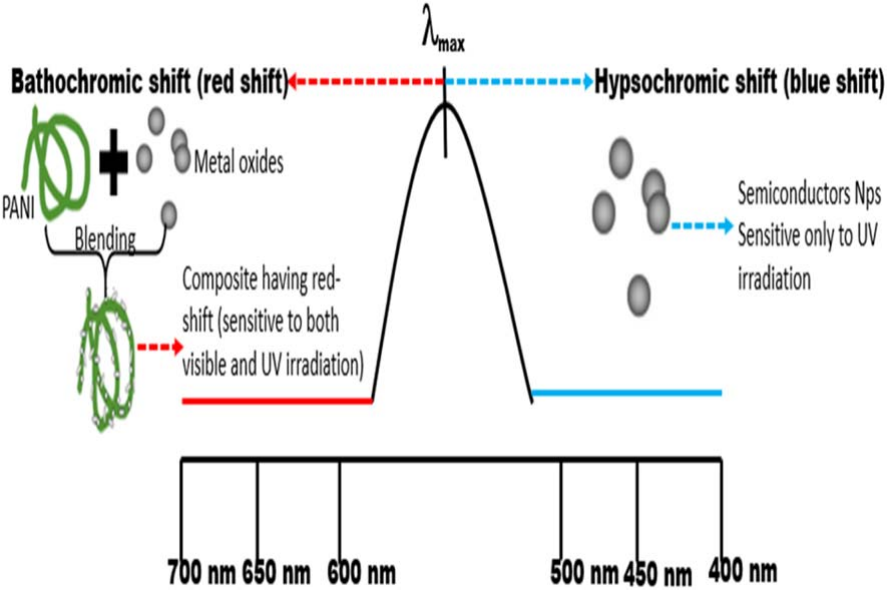
Band shift mechanism of pure semiconductor and PANI composites.

Thus, the fabrication of composites *via* the blending of PANI with semiconductors causes a band shift from hypsochromic (blue) to bathochromic (red shift) (Fig. 6), lowering the bandgap energy and the sensitivity of the composites to irradiation in the visible region of the electromagnetic spectrum.^5,59,72^ Also, Bouziani *et al.*^73^ reported that the presence of PANI in the composite enhances the fast separation and transfer of photogenerated electrons and holes, which improves the degradation efficiency. Other notable impacts of PANI include improving the functional properties and reactivity of the photocatalyst and the modification of the surface morphology of the composites, besides appreciable thermal and chemical stability.^58,74^ These features enhance the significant adsorption capacity of the catalyst when in contact with dye molecules in the effluent, establishing various bonding interactions such as van der Waals and electrostatic bonding, which facilitate dye degradation.^13,43^ Also, due to the effective anchoring of the materials along the polymeric network, the leaching of the catalyst and the rate of catalyst deactivation are reduced owing to the presence of more active site in the composite blend compared to the pure semiconductor material.^13^

## 3 Properties and performance of fabricated PANI composites in dye photocatalysis

The properties of fabricated PANI composites used as photocatalysts in the remediation of dye-laden effluent originate from the respective properties of their constituents, formulation, and generally the fabrication techniques, as highlighted in Table 1. Also, the properties of these catalysts dictate their corresponding performance in the mineralization of dye-laden effluent. These vital properties are elucidated *via* multiple characterization techniques, which are predominantly microscopic and spectroscopic. The different instruments that are available with unique principles and sample preparation, together with specific information on the fabricated photocatalyst are summarised in Table 2. The microscopic techniques involve the interaction of light or beams of an electron with matter to access properties such as size, distribution/dispersion in the solvent, degree of aggregation/agglomeration and morphology of nano polymeric composite photocatalysts.^75,76^ Alternatively, spectroscopic techniques elucidate the features of fabricated PANI composite photocatalysts based on the interaction of these nanomaterials with electromagnetic radiation from photon sources.^20,77,78^ This is done by quantitative assessment of the difference in the energy of their molecular energy levels, which are different for atomic and molecular structures.^20,79^ The use of the multiple instrumental techniques provides a wide spectrum of information on the properties of the fabricated material, which is in tandem with their performance in the degradation of dyes in effluent, as highlighted in Table 3. Table 1 indicates the various fabrication processes for catalyst composites, whereas the results in Table 3 indicate that the functional impacts of PANI in the blend, fabrication techniques, wt/wt% ratio of the composite constituents and nature of other materials equally determine the resultant properties and overall performance of the photocatalysts in dye photocatalysis.^4,16,80,81^

**Table 2:** Summary of the theory, principle, information and limitations of spectroscopic and microscopic instruments

Instruments	Theory/principle	Information	Advantages	Limitations	References
Fourier-transform infrared (FT-IR)	Works by vibrational spectroscopy	Determination of functionality of molecules	High sensitivity in parts per million (ppm)	Low resolution (under 20 mm)	17, 20, 82 and 83
	It involves the passage of monochromatic infrared radiation through a sample analyte, leading to the vibration of bonds between atoms based on their distinct level of energy absorption. This gives distinct absorption peaks, which correspond to the frequency of vibration of the molecule	Predict possible chemical reactions and bonding linkages	Fast analysis time (in seconds)	Inability to detect molecules of two identical atoms	
		Used to determine the analyte purity		Inability to analyze aqueous samples	
		Access the compatibility of the sample with a solvent		Requires sample preparation	
Raman spectroscopy	Similar in principle to FT-IR; however, its mode of operation is by inelastic scattering of light as it interacts with the molecular vibrational modes. This transfers some molecular vibration energy into light, resulting in a different wavenumber after its interaction with samples, giving distinct peaks in its spectra	Elucidation of functional groups of molecules	No sample preparation	Possible release of fluorescence light from the sample, causing background noise	83 and 84
		Information on crystal structure and chain orientation of polymers, hybridization and its composites	Highly sensitive to the detection and quantification of functional groups	Low Raman signal for polar molecules	
		Quantitative estimation of change in sample thickness		Profile depth of materials is limited to 0–25 μm	
				It is time-consuming	
X-ray diffraction (XRD)	Involves the incidence of monochromatic ray on the sample where the diffraction variation angle (2*θ*) existing at the sample surface is accessed *via* Bragg's equation	Structural elucidation of materials	Requires no sample preparation	Inability to interact with lighter elements and less sensitivity to heavy elements	83 and 85
	The diffraction is based on constructive interference of monochromatic X-rays sample morphology	Access crystal structure and distance between materials	Ability to detect a wide range of crystalline compounds	Most accurate only for large crystal structures	
				The possibility of overlapping peaks complicates the analytical study	
X-ray photoelectron (XPS)	X-ray beam incident on the sample leads to the release of electrons and kinetic energy. The detector quantifies the unique energy for each element as distinct binding energy	It identifies and quantifies elements	Very sensitive surface characterization for nanomaterial	Requires special sample preparation	86
		Determination of hybridization and oxidation states of elements		Its detection limit is 1000 ppm	
X-ray fluorescence (XFS)	Excitation of sample electron by incident X-ray beam causes an electron to be dislodged from the inner orbital shell of an atom, creating a vacant space. The space is filled by another electron, which drops with a high-energy orbital shell, bringing about a unique fluorescent X-ray for each element	Quantifies elemental composition of materials in wt%	Fast	The information given is only for the total element, not the ions	87 and 88
			Most samples do not require preparation	Inability to detect H_2_, O_2_ due to weak fluorescent X-ray	
Energy-dispersion X-ray spectroscopy (EDS)	Involves electron movement to fill the vacant site, which leads to the emission of X-rays to balance the energy difference between the electrons. When the rays hit the detector, created pulses are converted to a voltage by an analyzer in which the voltage is proportional to the energy of the X-rays	Detection and quantification of the elemental composition of a sample	Very fast	Sample preparation required	89
		Gives information on the mass fraction of each element		Samples need to be conductive	
		Use to monitor elemental changes in the polymeric composite photocatalyst during its fabrication		Limitation in detecting light elements	
				Cannot quantify ions	
Nuclear magnetic resonance (NMR)	Transmission of a radio wave to a spined sample placed between two powerful magnets, leading to the excitation of the nuclei of the sample atom. This excitation produces a resonance, which is detected by the receiver	It is applied in the analysis of the structural and functional group of samples	Low background noise	Requires sample	83
		Identifies chemical moieties and conformational state	Ability to detect polar molecules	Paramagnetic elements have less NMR signal	
		Can investigate nanocomposites, their miscibility and their degradation			
		Determines the chemical environment of elements			
Scanning electron microscopy (SEM)	Electron beam incident on the samples leads to the release of secondary electrons. These electrons are collected by the detector and analyzed as spectroscopic images with distinct magnification	Information on the surface morphology, pore and particle size of the fabricated nanocomposite photocatalyst	It has extended magnification up to one million	The instrument only analyses moisture-free solids	83, 90–92
			Its images are 3D, unlike TEM, which only reveals 2D images	Cannot measure the thickness of sample <10 nm due to its limited resolution	
				Require conductive samples for the appropriate imaging	
Atomic force microscopy (AFM)	It is based on the determination of forces between the tip of the probe attached to the cantilever and the sample. These interactions and deflection are converted to force, which can be quantified as a read-out image	Surface nano-profiling	More accurate in the measurement of mesoporous structures with pore size ranging from 2–50 nm	Requires more processing time	93–95
		Surface roughness/topology	Higher sensitivity force	Low field depth	
		Pore size distribution	Higher resolution		
			Requires no sample preparation		
Transmission electron microscopy (TEM)	Similar to SEM; however, transmitted electrons will form an image on a fluorescent screen, giving more details about the internal structure of the fabricated composite	Microscopic information on nanoparticles, structural features pores, 2D distribution and internal arrangement of the composite materials	Higher magnification and resolution of up to 50 million, making it more suitable	Images are 2D, and hence cannot show the topography data	83 and 96
				Sample staining is mostly required	
				Difficulty in the analysis of samples >100 μm	
Dark field microscopy (DFM)	This microscopic technique adopts a scattering-based imaging technique, whereby the elastically scattered light from a sample entering the objective forms the image, while incident light is blocked	It gives information on the resonant wavelengths of nano or metallic materials with high efficiency	Has high image contrast for nanostructural materials	High sensitivity to optical alignment makes its use difficult	97 and 98
				Requires high-intensity light	
UV-visible spectroscopy	Uses ultraviolet and visible light in the wavelength range between 200 and 780 nm to interact with samples. The concentration of the sample is a function of its interactive absorbance or transmittance of incident light using beer lambert's law	Give a quantitative measurement of the absorption concentration of a sample	A sensitive molecular spectroscopic method	May require sample preparation	99 and 100
				Quantifies one sample at a time	
				Spectrophotometers that work in non-visible range of the electromagnetic spectrum, such as those presented, which use expensive elements like diode arrays and charged couple devices (CCD) as sensors in the detecting stage	

**Table 3:** Performance and properties of PANI composite photocatalyst based on instrumental characterization^a^

S/N	Nano polymeric composite	Fabrication technique	Dye pollutants	Required time (h)	Irradiation source	Experimental conditions	Microscopic overview	Spectroscopic overview	Performance	Reference
1	PANI–TiO_2_	*In situ*	MB	6	Vis-lamp	5 mM, 4% dosage	Spherical dispersed TiO_2_ anchored on the polymeric chain by SEM and TEM	The crystallite size of 16.27 nm by XRD, lowered band gap of 3.15 eV by UV-spec	99%	110
2	PANI/TiO_2_	Self-assembly	MO	3	40 W high-pressure mercury lamp	0.5 mM, 7.0 pH	Aggregated nano spherical and tubular morphology by SEM and TEM	No change in the FT-IR band before and after	94.2% and 97.2%	10
			Orange II							
3	PANI/TiO_2_	*In situ*	RB5	3	150 W UV lamp	10 mg L^−1^, 15 mg dosage	Nanotubes encapsulated with uniformly dispersed TiO_2_ particles by SEM and TEM	Reduction in band gap to 2.10 eV by the UV-spec	96%	111
								Reduction in peak intensity on the addition of PANI by XRD spectra		
								Weaken conjugated system identified by Raman spec in the mix		
4	PANI–TiO_2_	*In situ*	MB	2	110 W high-pressure sodium lamp	10 mg, 10 mg L^−1^	The network-like polymeric structure identified by SEM with dispersed TiO_2_ particles on the polymeric network	XPS reveals the atomic concentration of Ti and O on the surface of PANI	81.7%	112
								UV-spec reveals improved higher light absorption with the addition of PANI		
5	PANI–TiO_2_	Sol–gel	Tartrazine	2	10 W low-pressure mercury lamp	5 mg, 10 mg L^−1^	Sponge-like shape material from SEM with evenly distributed spots of TiO_2_ from the TEM	XRD reveals unaffected high crystallinity by the peak intensity of rutile and anatase indicating surface deposition of TiO_2_ unto the polymer	99%	113
								EDX reveals even elemental distribution on the surface of the polymer		
6	PANI–TiO_2_	Electrospinning and dispersion	MB	5.42	Sunlight	100 mg, 10 mg L^−1^	SEM and TEM reveal micro fibrous compact TiO_2_ together with PANI nanowire with TiO_2_ having increasing roughness on the addition of PANI	Band shift of PANI on the addition of TiO_2_ identified by FT-IR	91.5%	114
							High pore size and reduced agglomeration	UV-vis reveals the extended light absorption of TiO_2_ to the visible region		
7	PANI/TiO_2_/Cotton	*In situ*	Rhd-B	3	300 W Xenon arc lamp	1.0 × 10^−5^ M	A pinstriped and spherical particle of PANI on fiber with small agglomerated TiO_2_ by SEM and TEM	More –OH group indicated with high intensity by FT-IR, higher binding energy identified with XPS	87.7%	115
8	PPy-PANI/TiO_2_	Suspension	4-Nitrophenol	2	300 W Xenon lamp	0.15 g, 10 mg L^−1^, pH = 6.08	Micropore structure spotted with TiO_2_	FT-IR indicates the synergistic bonding between the materials, forming composites	99%	107
								UV-spec indicates a lower band gap for the composites		
								XRD reveals average crystallite size with lower aggregation for the composites		
9	PVA/PANI/TiO_2_	Self-assembly	MB	2	100 W mercury lamp	10 mg, 25 mg L^−1^	Fabricated hydrogels with open channels by SEM	Intense and broad peaks identified in the addition of PANI	78.7%	116
			MO				Coral-like dendrite morphology by TEM	Narrow peaks of PVA, which was later dominated by TiO_2_ and PANI	84.5%	
								FT-IR relates the established bonding arrangement between PVA and PANI with intense and broad absorption bands in the addition of TiO_2_		
10	PANI/Fe–TiO_2_	Mixing	MB	2.5	30 W UV lamp	10 mg L^−1^	SEM and TEM revealed microstructural crystalline well-ordered aggregation of TiO_2_ and Fe–TiO_2_ unto the polymeric network	FT-IR reveals the bonding linkage between Fe and TiO_2_ and PANI–Fe	28%	117
								XRD reveals the peak pattern of TiO_2_ with the titanium ion substituted with Fe in the crystal lattice		
								UV spec revealed lowered band gap of TiO_2_ from 3.18 eV to 2.71 eV		
11	PANI/TiO_2_/SiO_2_	*In situ*	MO	2	500 W Xenon arc lamp	1.5 mg L^−1^	Spherical TiO_2_ and SiO_2_ nano fibre coated with PANI powder reported by SEM and TEM	Increased peak intensity on the addition of SiO_2_ by XRD	87%	108
								UV spec revealed the photosensitizing attribute of PANI in addition due to the observed lower bandgap		
12	TiO_2/_Nb_2_O_5_/PANI	Hydrothermal	MB	4	300 W UV lamp	20 mg L^−1^, pH 7	Spherical particles of TiO_2_/Nb_2_O_5_ lesser aggregation with clustered growth on the addition of PANI by SEM	Reduced crystallites size reported after composite fabrication by XRD	99%	8
			MO				TEM indicates the agglomerated grain structure with a spherical shape on the polymeric surface	UV spec revealed lowered band gap from 3.2 eV to 2.75 eV	97%	
13	PANI–TiO_2_/rGO	*In situ*	Rhd-B	1.5	300 W Xe lamp	50 mg, 1 × 10^−5^ M	Wire-like morphology reported by SEM image with multiple fold rGO by TEM connected to polymeric network	XPS indicates the sp^2^ hybridization corresponding to the carbon of graphene in connection with PANI p–p stacking interaction of TiO_2_ and rGO by Raman spec	90.5%	34
								FT-IR relates the Ti–O–Ti linkages and skeletal vibration of TiO_2_/rGO		
14	PANI/ZnO	Arch discharge	MB	5	Sunlight	0.4 mg mL^−1^, 1 × 10^−5^	Uniform dispersion of ZnO on the polymeric matrix. TEM revealed ZnO particles enclosed in the polymeric nanorods of PANI	Reduced peak intensity on the addition of PANI by XRD	97%	36
			MG					Observed broad peak shift to higher wavelength on the addition of ZnO	99%	
15	PANI/SiO_2_	Sonochemical	MB	2		50 mg, pH 6.7	Observed flake-like shape of SiO_2_ anchored unto PANI network by SEM and TEM images	The broader peak of PANI becomes narrower with the addition of SiO_2_ from the XRD spec	74%	118
			BB					Observed disappearance of aromatic peaks due to formation from FT-IR	86%	
16	Ag–ZnO/PANI	*In situ*	MG	2	Visible light	0.2 g L^−1^, 0.2 g, pH 8	—	The XRD spectra showed the peak pattern of the wurtzite structure of Ag–ZnO with increased peak intensity on the addition of PANI	98.6%	101
								Observed band gap reduction from 3.2 eV to 2.87 eV and a red shift for Ag–ZnO and lower bandgap of 2.61 on the addition of PANI		
								FT-IR reveals the insertion of Ag–ZnO to the polymeric network from the spectra of the bond linkage		
17	Cu_2_O/ZnO-PANI	*In situ*	CR	0.5	100 W LED	0.05 g L^−1^, 100 mg, pH 6	SEM revealed the quasi-spherical morphology of the composites with high polydispersity	XRD plane pattern revealed the cubic structure of Cu_2_O and the hexagonal plane of ZnO with lower crystallinity of addition of PANI and lower crystallite size	95%	119
							HRTEM spherical nanoparticle of Cu_2_O dispersed on the flake-like ZnO anchored unto PANI network	XPS indicates the lower binding energies of the metal oxide in the addition of PANI		
								EDX revealed the elemental composition which is in tandem with the stochiometric ratio of corresponding elements		
								Raman spec relates the stretching vibration of polaronic structures of PANI and the peak pattern of the metal oxides similar to FT-IR		
19	2Dh-BN/PANI	*In situ*	MB	1.5	UV-source	20 mg and 10 mg L^−1^	SEM and TEM revealed the disc-like shape of the semiconductor and tubular shape morphology, which was because of the granular structure after the blend was formed	XRD reveals a well-ordered peak of 2Dh-BN which reduces the addition of PANI	93%	4
			MO					IR identified the redshift of the peak shift after doping	95%	

a MB: methylene blue; MO: methyl orange; CR: congo red; BB: brilliant blue; RB5: reactive blue 5; Rhd-B: rhodamine B; MG: malachite green; Spec: spectroscopy; 2Dh-BN: 2D hexagonal boron nitride.

For instance, the technique employed (*in situ* polymerization) in doping PANI with photon active metals such as Ag to form Ag–ZnO/PANI composites creates high photon absorption at the visible spectrum of electromagnetic radiation, resulting in a high photocatalytic performance of 98.6% for malachite green (MG).^101^Table 3 reveals that *in situ* polymerization techniques are the most prevalent due to the formation of composite blends with better self-organization, improved optoelectrical and conductive properties of the mix, and decreased aggregation of the nanoparticles because of the effective interfacial synergism, as revealed by the instrumental characterization.^69,102^

Also, the notable performance of the PANI–TiO_2_ blend recorded in Table 3 (99% and 96%) for MB and RB5 dye, respectively, is consistent with the reduced crystallite structure from the electron imaging and the lowered bandgap brought about by the incorporation of PANI. The impact of PANI in this blend limited the agglomeration and improved the photon-capturing potential of the composite up to the visible region.^46^ The other novel methods with a high performance for the degradation of MB, as indicated in Table 3, are electrospinning and dispersion, suspension, self-assembly, hydrothermal, arc-discharge, sonochemical, and sol–gel methods with the performance of 91.5, 99, 78.7, 99, 97, 74 and 99%, respectively. However, the preference for *in situ* techniques is due to their fast reaction rate, cost-effectiveness and ability to control the conditions for the formation of composite blends.^4,69,103^ In addition, *in situ* polymerization is a one-step technique for the fabrication of nano composite photocatalysts with beneficial attributes such as effective spatial distribution of associated nanomaterial in the polymeric matrix of PANI and higher interfacial strength, which contribute to lower interparticle spacing and improved optoelectronic potentials in composites.^69,104^ Furthermore, instrumental elucidation of the internal morphology of the materials *via* SEM and TEM revealed the well-ordered distribution of the nanoparticles, low degree of agglomeration and occurrence of surface modification *via* coupling of PANI with metal oxide semiconductors (TiO_2_, ZnO, SiO_2_, rGO and Cu_2_O) and metals such (Ag and Fe). For instance, Zarrin & Heshmatpour^8^ described the functional impacts of PANI in the blend forming TiO_2_/Nb_2_O_5_/PANI *via* SEM imaging. According to their results, the composites exhibited a spherical morphology, lower degree of agglomeration and larger surface area. Also, the presence of PANI in this hybrid composite serves as a physical barrier and conduction path, which are essential for the effective separation and transport of photogenerated electrons and holes and to hinder their possible recombination.^14,105^ The variation in morphological attributes of the composites such as wire-like morphology (nanowire) for PANI–TiO_2_/rGO, enclosed uniform dispersion (nanoparticle) for PANI/ZnO composites, flake-like (nanoflake) for PANI/SiO_2_, and quasi-nano spherical for Cu_2_O/ZnO-PANI described in Table 3 is a function of the nature, mode of fabrication and interactive synergism of PANI with the semiconductors in the mix.^13,33,106^

Likewise, the XRD elucidation of the nanocomposites, as shown in Table 3, demonstrated a reduction in peak intensity for most of the fabricated composites except for the PPy-PANI/TiO_2_ blend. This exception suggests the stability of TiO_2_ despite its interfacial coordination with PANI and polypyrrole.^107^ However, the predominant reduction in the peak intensity is due to the successful incorporation of the amorphous PANI in the well-ordered crystalline structure of the semiconductors used.^4^ This reduction in intensity is related to the reduction in the crystalline properties of the mix, which is associated with the reduction in crystallite size calculated using Scherrer's equation.^108,109^ It is worth noting that the reduction in the crystallite size is predominant in Table 3 except for PANI/Fe–TiO_2_. The XRD characterization of this composite showed the tetragonal lattice structure of titanium, which was altered *via* the substitution of titanium ions by iron ions, increasing the average crystallite from 19 nm to 20 nm and leading to a low efficiency of 28% for MB dye, as shown in Table 3. Hence, the altered peak intensity and pattern revealed by the instrument indicate the successful formation of a composite blend with either improved crystalline or amorphous properties, which influence its functional performance and chemical stability.^109^

The XRD data was compared with the EDX results to show the elemental composition of the composites, which is consistent with the stoichiometric ratio of the elemental constituent. Alternatively, XPS gives the relative binding energies and relates the hybridization based on the interaction of the coupled composite photocatalyst.^23,34^

Also, according to this, the FT-IR spectra revealed the functional properties, bonding sequences, linkages and spectra shift occurring in the composite molecules. Composites such as PANI/ZnO and PANI/TiO_2_ in Table 3 exhibit an observable shift in their characteristic peak, which is based on the interactive linkages between the PANI and metal oxides. This often results in the alteration of the electron densities and bond energies of PANI.^120,121^ A shift to lower wavenumbers indicates an increase in the electron density of the PANI chains.^122^ This action is desirable in dye photocatalysis with the nanocomposite catalyst and indicates the efficient insertion of the semiconductor into the macromolecular network of PANI.^122^

Also, a notable redshift was reported for many of the composites in Table 3 by UV spectroscopic elucidation. Shahabuddin *et al.*^4^ reported that this shift can be due to van der Waals linkages, π–π or electrostatic interaction. At this point, the positively charged polymeric backbone establishes a synergic interaction with the compositing materials during fabrication.^72,123^ This interaction enhances the light absorption propensity of metal oxides such as TiO_2_ in the visible region of the electromagnetic spectrum.^124^ For instance, for the Ag–ZnO/PANI nanocomposite in Table 3, its first absorption band arises from the π–π* electron transition in the benzenoid segments, while the second and third absorption bands are related to the doping and formation of polarons, respectively.^14,125^ In addition, the reduction in the bandgap evaluated from the spectra shows the interactive mechanism and improved optical absorptivity of the mix especially composites such as PANI/TiO_2,_ PANI/Fe–TiO_2_, TiO_2/_Nb_2_O_5_/PAN and Ag–ZnO/PANI.

## 4 Selected instrumental characterization of fabricated PANI composite photocatalysts

### 4.1 Selected microscopic overview

**Fig. 7:**
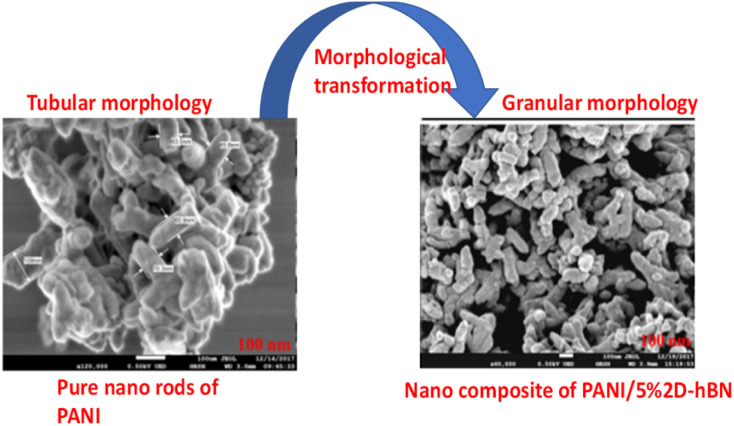
FE-SEM image of pure PANI and composite of PANI with 5 wt/wt% of 2Dh-BN. Image adapted from Shahabuddin *et al.*^4^ Reproduced with permission from, Elsevier.

A microscopic overview on the functional impacts of PANI in the composite morphology studied *via* FE-SEM and TEM techniques gives the descriptive morphological property and surface topology of the fabricated nanocomposites at various wt/wt%. Shahabuddin *et al.*^4^ studied the degradation of methylene blue and methyl orange using a polymeric composite fabricated from polyaniline and 2D-hexagonal boron nitride. The FE-SEM and TEM images are shown in Fig. 7. The FE-SEM micrograph reveals the tubular morphological characteristics of the pure polyaniline. However, after the addition of 2D h-boron nitride *via in situ* polymerization, the fabricated nanocomposites transformed into granular structures, as shown in Fig. 7. The altered surface modification after fabrication was equally described by the FE-SEM image of the polyaniline/TiO_2_ photocatalyst studied by Gilja *et al.*^21^ and Aamir *et al.*^126^ in the synthesis and characterization of polyaniline/Zr–Co-substituted nickel ferrite nanocomposites for the photodegradation of methylene blue.

**Fig. 8:**
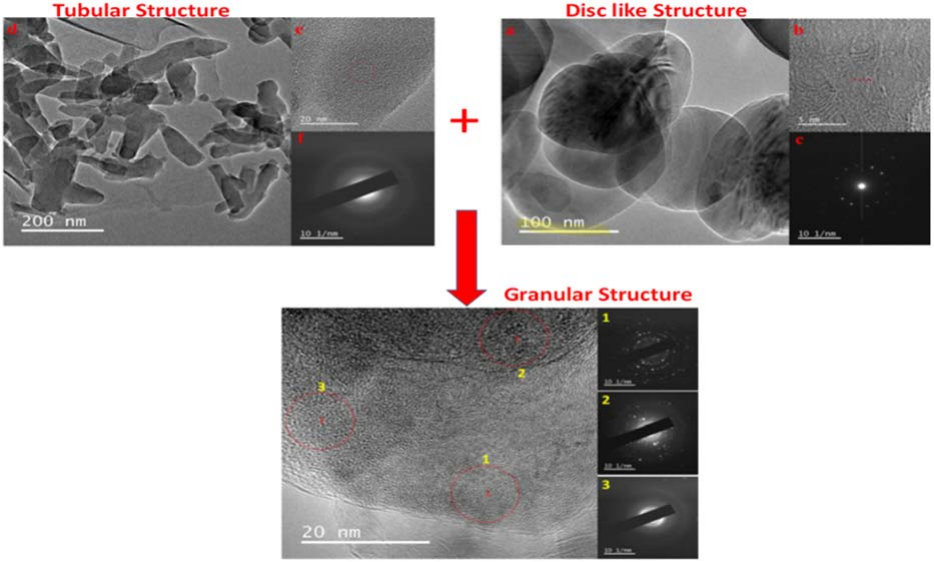
(a and b) TEM images of h-BN nanosheets (c) SAED pattern for h-BN (d and e) TEM images of PANI nanotubes and (f) SAED pattern for PANI nanotubes Image adapted from Shahabuddin *et al.*^4^ Reproduced with permission from Elsevier.

Mitra *et al.*^60^ also reported the microscopic assessment of a composite consisting of aluminum-doped zinc oxide/polyaniline (AZO/PANI), where the morphological features of PANI appear as nanorods, which are similar to the commonly used conventional catalysts (titanium dioxide) studied by Egerton.^127^ It is worth noting that the arrangement of these nano-rods of PANI enhances the ease of the formation of the composite mix, which provides active sites for adsorption–desorption before the photodegradation of adsorbed dye molecules.^128,129^ Ameen *et al.*^43^ studied the morphology of novel graphene/polyaniline nanocomposites and their photocatalytic activity toward the degradation of rose Bengal dye. According to the microscopic FE-SEM study, the tubular structure of PANI transformed into a layered sheet morphology with an average thickness of several hundred nanometres after the fabrication of the composite. Additionally, the TEM result of PANI and its corresponding composite (2D-hBN and PANI-2Dh-BN) by Shahabuddin *et al.*^4^ gives a two-dimensional image of the tubular structure of PANI (Fig. 8).

**Fig. 9:**
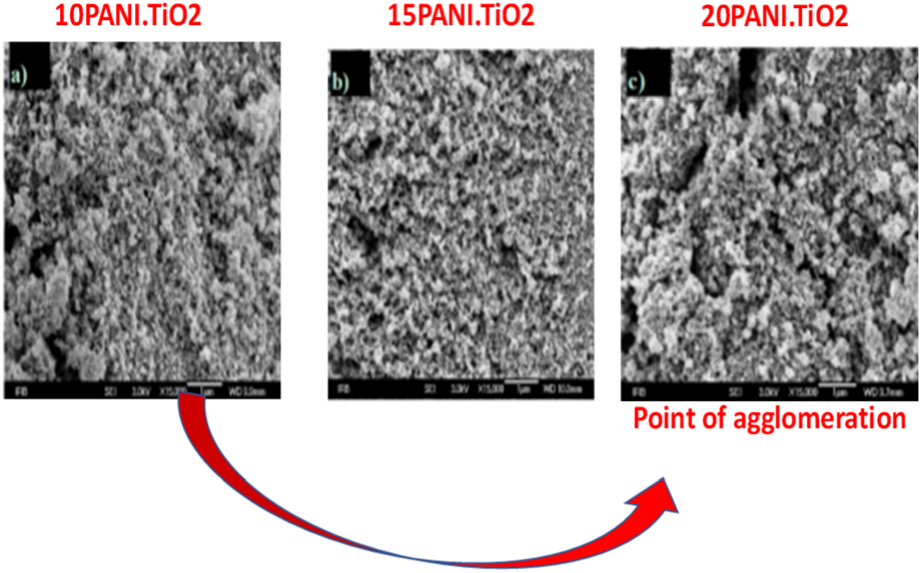
SEM micrographs of (a) 10PANI/TiO_2_; (b) 15PANI/TiO_2_ and (c) 20PANI/TiO_2_ composites (magnification ×15 000). Image adapted from Gilja *et al.*^21^ Reproduced with permission from MDPI.

This implies that the functional interaction of PANI with the disc-like structure of 2D-hBN (Fig. 8) enhances the surface modification of the composite, transforming it into a granular structure.^4^ A similar surface transformation was reported by Aamair *et al.*^126^ from the SEM study of polyaniline/Zr–Co-substituted nickel ferrite (NiFe_1.2_Zr_0.4_Co_0.4_O_4_) nanocomposite photocatalyst, leading to a high degradation efficiency of 97% for methylene blue dye. The image in Fig. 8 indicates the amorphous rod-like structure of polyaniline compared to the well-ordered disc-like crystalline structure of the 2Dh-BN, which enhances its size and optoelectronic reactivity.^4,130^ These microscopic techniques are capable of revealing the point of agglomeration during the fabrication of the composite, as described in Fig. 9. Gilja *et al.*^21^ and Shahabuddin *et al.*^4^ revealed that pure PANI does not undergo agglomeration due to its smaller aggregate sizes from its lower inverse barrier. This effect is due to the ability of aniline molecules to create a barrier effect, which lowers the aggregation of polyaniline.^131^ However, the FE-SEM study by Chatterjee *et al.*^132^ on a polyaniline-single-walled carbon nanotube composite showed that PANI undergoes agglomeration at a high concentration of aniline, similar to Fig. 9. Meanwhile, the interaction of PANI with the single-walled carbon nanotube after the fabrication of the composite hindered the formation of agglomeration, while increasing the surface area of the blend.^132^

The agglomeration revealed in Fig. 9 consequently alters the synergistic effect with available binding sites, limiting the dye-degrading propensity of the composite photocatalyst.^4^ Hence, microscopic elucidation projects the point of agglomeration and reveals the morphological transformation that occurs during the fabrication process before dye photocatalysis.^126^

### 4.2 Selected spectroscopic overview

#### 4.2.1 Fourier transform infrared spectroscopy (FT-IR)

**Fig. 10:**
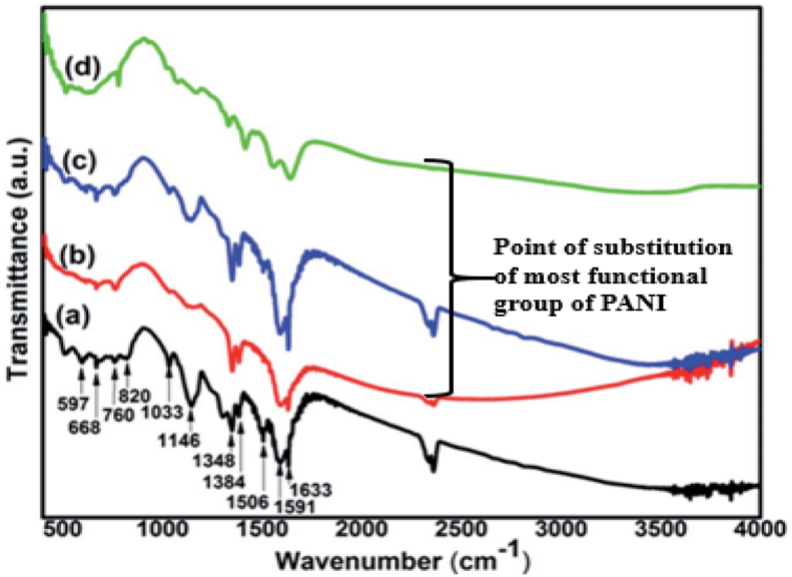
Pure PANI (a); PANI-1% SWCNT (b); PANI-2% SWCNT (c); and PANI-4% SWCNT (d). Image adapted from Chatterjee *et al.*^132^ Reproduced with permission from RSC Advances.

One of the vital spectroscopic instruments commonly used to elucidate the functional groups of fabricated photocatalytic nanocomposite is the FT-IR spectrometer.^133^ The characterization using this instrument provides information on molecular structure, chemical bonding and molecular environment, which suggests the expected chemical interaction, dye adsorption and degradation mechanism during photocatalysis.^133,134^Fig. 10 reveals the spectra resulting from the application of this instrument for comparative assessment of pure polyaniline and its corresponding composite. The fabricated nanocomposite consisted of PANI blended with single-wall carbon nanotubes (SWCNT) at 1% SWCNT (b), 2% SWCNT (c) and 4% SWCNT (d). According to this figure, peaks such as 820 cm^−1^ vividly describe the aromatic C–H bending for the 1,4 di-substituted benzene rings, while the peaks at 1348 cm^−1^ and 1384 cm^−1^ correspond to the C–N stretching of the secondary aromatic amine. The stretching indicates the stronger bonding interaction of the functional groups in PANI with the SWNT coupled with the respective blue shift to 1416 cm^−1^, 1557 cm^−1^, and 1643 cm^−1^ from 1384 cm^−1^, 1506 cm^−1^ and 1633 cm^−1^, respectively. Also, the peaks located at 1633 cm^−1^ and 1560 cm^−1^ confirmed the presence of the C=C quinoid ring and benzoid, respectively.^132,135^ As shown in Fig. 10a–c, Chatterjee *et al.*^132^ reported that reveals the chemical interaction of SWCNT with PANI at different reaction sites. Similarly, this action was observed by the FT-IR study of the PANI/nano-SiO_2_ composite and PANI-MWCNT, where the bond strength and the bond weakness of the formed composite were a function of the wavenumber.^136,137^

Furthermore, Yang *et al.*^138^ applied FT-IR for the characterization of graphene oxide and polyaniline (GO/PANI) nanocomposites, where the absorption bands of PANI decreased in the spectra of the GO/PANI composites. This indicates the substitution of most of the functional groups present *via* chemical reduction.^138^ Sarmah and Kumar^139^ observed a shift in the C–N stretching of the benzenoid unit from 1296 to 1315 cm^−1^ when a composite of PANI is formed with TiO_2_ (PANI/TiO_2_). The shift to a higher wavenumber described the impact of the chemical interaction of N atoms from C–N in the polymer chain with the O atoms of TiO_2_, which suggest electron delocalization. Also, Shahabuddin *et al.*^4^ observed a similar band shift when a composite of polyaniline and 2D hexagonal boron nitride was fabricated. Their study confirmed that the band shift may be due to weak interactions such as van der Waals attraction between the positively charged PANI backbones and the h-BN molecules. King *et al.*^136^ and Li *et al.*^137^ reported that the photon-capturing potential of the catalyst composite is based on the functional interaction of PANI with the semiconductor, which strengthens the composite reactivity during photon irradiation for dye degradation.

#### 4.2.2 X-ray diffraction (XRD)

**Fig. 11:**
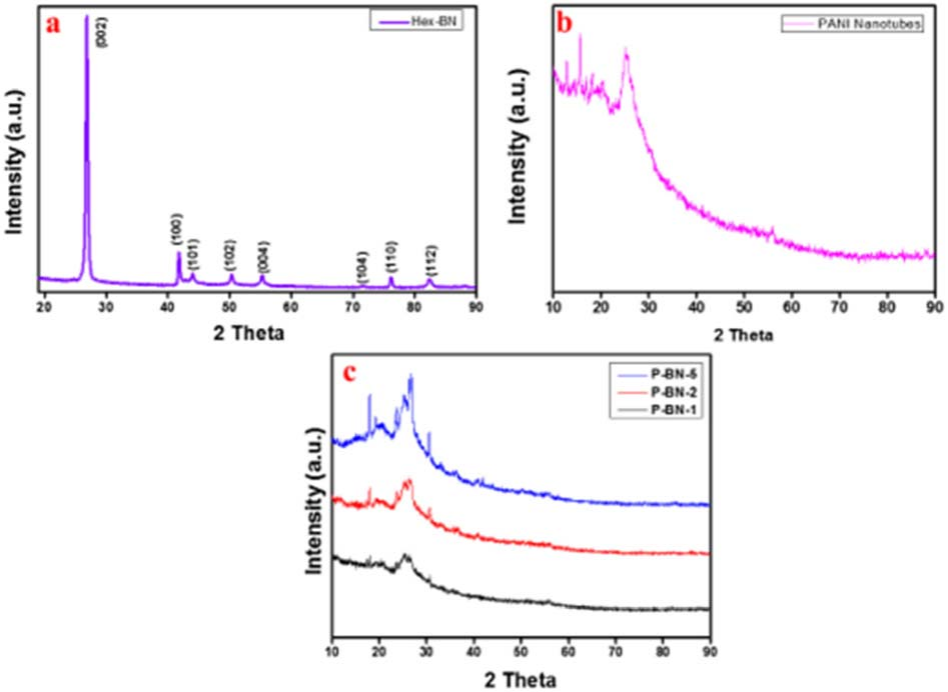
XRD patterns of (a) h-BN nanosheets, (b) PANI nanotubes and (c) h-BN nanosheet-doped nanocomposites. Image adapted from Shahabuddin *et al.*^22^ Reproduced with permission from Elsevier.

The use of this instrumental technique holds great importance in the analysis of fabricated composites regarding their crystalline and amorphous orientation, size, shape and internal stress of small crystalline regions. However, the measurement of this parameter depends on the peak position, width and intensity.^140,141^ Shahabuddin *et al.*^4^ studied the orientation of pure polyaniline, 2Dh-boron nitride (2D-hBN) semiconductor and a composite comprised of these two materials at different weight percents (Fig. 11). The highly ordered structural pattern of the semi-conductor (2D-hBN) was reflected by the peaks with 2*θ* values of 26.80°, 41.70°, 42.95°, 50.20°, 55.28°, 71.41°, 75.98° and 82.27, corresponding to the expected crystallographic planes of pure h-BN of (0 0 2), (1 0 0), (1 0 1), (1 0 2), (0 0 4), (1 0 4), (1 1 0), and (1 1 2), respectively, according to JCPDS file number 01-073-2095.^142,143^ Furthermore, the pure conductive PANI exhibited peaks at 15.76°, 20.35°, and 25.25°, showing a polycrystalline structure; however, the regions with broader peaks are the amorphous potion of the polymers.

The peak value was also affirmed by Chatterjee *et al.*^132^ in their comparative study of PANI and SWNTs. The broad peak of PANI was equally observed for PANI and PANI/ZrCo-substituted nickel ferrite composite. According to their study, the prominent peaks of the conducting polymer were located at 20.4°, 25.4°, and 28.2°. However, the altered structural pattern was due to the reported extra peaks at 35.83°, 37.20°, 43.5°, 50.1°, 54.3°, 57.2°, 63.0°, and 74.8° when matched with the standard pattern.^126^ The intense peaks commonly observed at around 20.35° and 25.25° can be linked to the repetitive sequence of the benzenoid and quinoid rings, respectively in the PANI backbone.^139^ However, for the composite mix at different wt%, the sharp decline in the peak value and the increasing intensity, as shown in Fig. 11, are related to the successful interaction of the semiconductor with the highly amorphous homopolymer.^4^ This is because the semiconductor used in its pure state exhibits an appreciable level of crystallinity, as deduced from the peak intensity in Fig. 11. However, with the addition of the amorphous PANI, the peak intensity decreased due to the amorphous interaction of the macromolecule network (PANI) with the well-ordered molecules of the semi-conductor.^4^ Aamir *et al.*^126^ suggested that reduction in peak intensity is directly proportional to the increase in the concentration of PANI, which functionally influences the bandgap tunability and photocatalytic performance. Furthermore, Sarmah and Kumar^139^ studied the fabrication of a PANI/TiO_2_ composite for the remediation of dye effluent. The result of the XRD spectra for PANI in the composite mix of PANI/TiO_2_ did not exhibit changes in peak positions and shapes compared to the TiO_2_ rod. This observed action illustrates the mere attachment of PANI to the surface of the semiconductor rod.^139,144^ This action could be due to the method employed for the fabrication of the composite or the experimental conditions set during the fabrication process.^13^ The orientation of the formed composite affects the band gap tunability, which indicates its photon-capturing propensity during photocatalysis of dye effluent.^4,13,43^

#### 4.2.3 UV-visible spectroscopy

**Fig. 12:**
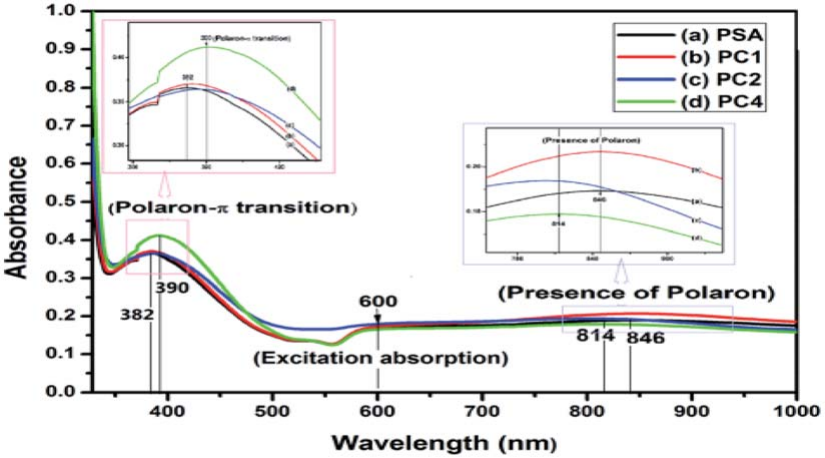
Normalized UV-vis spectra of (a) PSA, (b) PC1, (c) PC2 and (d) PC4. Where, PSA is acid doped PANI without SWCNT-single-wall carbon nanotube; while PC1 (1% SWCNT composition), PC2 (2% SWCNT composition) and PC4 (4% SWCNT composition) with polyaniline. Image adapted from Chatterjee *et al.*^132^ Reproduced with permission from RSC Advances.

The spectra of materials can be measured in the wavelength range of 800 nm to 2500 nm using a ultraviolet spectrophotometer (UV), visible spectrophotometer (vis), and near-infrared spectrophotometer (NIR).^99,145,146^ Composite quantification with these instruments uses ultraviolet and visible light in the wavelength range of 200 and 780 nm.^99^ Studies show that these instruments induce analyte electronic transitions such as π → π*, n → π*, n → σ*, δ → δ and charge transfer transitions.^5,147^ However, the predominant transitions occurring during the spectroscopic investigation of an emerging polymeric composite of PANI are π → π* (molecules with π bonds) and n → π* transitions, involving lone pair electrons that exist on heteroatoms such as oxygen and nitrogen atoms.^99,148^ These transitions generate spectra whose readout gives vital information on optoelectronic stability and the response of the nanocomposite, calculated bandgap, and synergic interaction of polymers with other materials.^148^ As shown in Fig. 12, the assessment of a PANI composite by Chatterjee *et al.*^132^ revealed that two distinct peaks appeared at 373 and 417 nm, which is consistent with the excitation characteristics of the quinoid ring and the π–π* transition of the benzenoid ring.^124,149^ However, the reduction in peak intensity with the increasing addition of nickel ferrite NPs to form composites distinguishes the optoelectronic behaviour and unique band gap of the composites compared to pure PANI. This resulted in an increase in the calculated band gap value from 2.2 eV for pure PANI to 2.4 eV with the addition of nickel ferrate.^132^ Furthermore, an investigative assessment of PANI and graphene oxide composite using UV spec by Yang *et al.*^138^ showed a similar transition of the quinoid, while the n–π* transition justified the presence of heteroatoms (oxygen) in the functional group of graphene oxide.^148^ The new absorption at distinct wavelengths identified from the spectra revealed the formation of new composites with distinct band gaps.^4,150^ In the instrumental elucidation by Sarmah and Kumar,^139^ they further observed a characteristic peak at 430 nm, indicating the p band-polaron band of protonated PANI chains, while the peak at 840 nm indicates the polaron band π* of doped PANI.^25^ The features revealed by the spectra indicate the presence of a single broad polaronic band deep in PANI stabilized by the coulombic interactions, dielectric screening and local disorder in the polyaniline.

#### 4.2.4 X-ray photoelectron (XPS)

The X-ray photoelectron spectroscopic technique is another vital instrumental technique that relates the elemental composition to the binding energies, valence states and chemical environment of the constituent elements forming composites.^132^

**Fig. 13:**
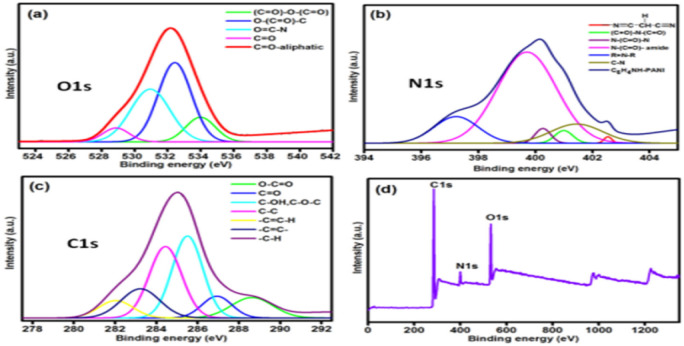
High-resolution O 1s, N 1s, and C 1s (a–c) spectra; and (d) full XPS survey of polyaniline–nitrogen-doped carbon dot nanocomposite. Image adapted from Maruthapandi *et al.*^151^ Reproduced with permission from MDPI.

Although similar to energy-dispersive X-ray spectroscopy (EDX), EDX is strictly applied for elemental composition and its respective abundance. Fig. 13(a–c) shows the high-resolution XPS spectra of O 1s, N 1s, and C 1s and comprehensive XPS investigation PANI composites (polyaniline–nitrogen-doped carbon dot nanocomposite).

The spectra show the characteristic peaks of the elemental composition of the composite under investigation, indicating the presence of C, N, and O and the elemental interaction in the composite as a function of their respective binding energies. Equally, the XPS investigative elucidation of graphene/polyaniline showed a unique binding energy peaks at approximately 284.4 eV, 397 eV and 529 eV for C 1s, N 1s and O 1s, respectively. These deconvoluted peaks reveal the interaction and bonding sequence between the conducting polymer (PANI) and the semiconductor graphene, forming composites.^43,151^ The peak value at C 1s at 284.4 eV also indicates the sp^2^ C of graphene, Gr, and the C=C conjugation of the benzenoid ring of the polymer, showing the interactive mechanism of protonation of imine and amine during the fabrication of the composite.^43^ Similarly, Chatterjee *et al.*^132^ reported the binding energy of 285.5 eV and 530.5 eV for C 1s and O 1s related to the pure PANI SWNT having 283.9 eV and 283.5 eV, respectively, while that of nitrogen (N 1s) was approximately 399.2 eV, which indicates the quinoid amine in the backbone of PANI, while the positively charged nitrogen is indicated by the higher peak of 401.2 eV, representing a protonated amine.^137,152^ This shows the synergistic interaction and formation of partial hydrogen bonding between the cationic nitrogen radical and the carboxylate group of the graphene moiety.^153–155^

## 5 Conclusion and future prospects

In this review, we revealed the functional impacts of PANI in the fabrication of composite catalysts for dye photocatalysis *via* instrumental outlook. It was revealed that particle agglomeration, poor surface area, porosity, frequent electron–hole recombination, and large bandgap limiting photon capture in the visible region are the major limitations in the photocatalytic treatment of dye-laden effluent. Considering this limitation, it is necessary to incorporate the conducting polyaniline, which when characterized *via* microscopic and a spectroscopic technique, creates functional attributes of improved surface morphology and topology, reduction in electron–hole pair, lowering of the band gap and impedes the formation of agglomeration by the nanocatalyst. Also, the study indicated the techniques for the fabrication of composites greatly influence the functional attributes of PANI and the corresponding properties of the mix, while *in situ* polymerization was identified as the most effective based on its excellent interfacial synergism. However, the future outlook involves the use of instrumental characterization to effectively study the bond mechanism of the fabricated composites and their interaction with dye molecules in the effluent. Furthermore, it is necessary to quantitatively determine the limits of PANI concentration required in the mix, beyond which may result in the possible agglomeration of the catalyst and ineffective recovery and reuse for other treatment runs.

## Conflicts of interest

The authors declare an absence of competing financial interests in personal relationships that could influence the work reported in this paper.

## Supplementary Material
